# GrabBlur - a framework to facilitate the secure exchange of whole-exome and -genome SNV data using VCF files

**DOI:** 10.1186/1471-2164-15-S4-S8

**Published:** 2014-05-20

**Authors:** Björn Stade, Dominik Seelow, Ingo Thomsen, Michael Krawczak, Andre Franke

**Affiliations:** 1 Institute of Clinical Molecular Biology, Christian-Albrechts University of Kiel, Schittenhelmstraße 12, 24105 Kiel, Germany; 2 NeuroCure Clinical Research Centre, Charité - Universitätsmedizin, Charitéplatz 1, 10117 Berlin, Germany; 3 Institute of Medical Informatics and Statistics, Christian-Albrechts University of Kiel, Brunswiker Straße 10, 24105 Kiel, Germany; 4 TMF - Technology and Methods Platform for Networked Medical Research, Charlottenstraße 42, 10117 Berlin, Germany

## Abstract

**Background:**

Next Generation Sequencing (NGS) of whole exomes or genomes is increasingly being used in human genetic research and diagnostics. Sharing NGS data with third parties can help physicians and researchers to identify causative or predisposing mutations for a specific sample of interest more efficiently. In many cases, however, the exchange of such data may collide with data privacy regulations. GrabBlur is a newly developed tool to aggregate and share NGS-derived single nucleotide variant (SNV) data in a public database, keeping individual samples unidentifiable. In contrast to other currently existing SNV databases, GrabBlur includes phenotypic information and contact details of the submitter of a given database entry. By means of GrabBlur human geneticists can securely and easily share SNV data from resequencing projects. GrabBlur can ease the interpretation of SNV data by offering basic annotations, genotype frequencies and in particular phenotypic information - given that this information was shared - for the SNV of interest.

**Tool description:**

GrabBlur facilitates the combination of phenotypic and NGS data (VCF files) via a local interface or command line operations. Data submissions may include HPO (Human Phenotype Ontology) terms, other trait descriptions, NGS technology information and the identity of the submitter. Most of this information is optional and its provision at the discretion of the submitter. Upon initial intake, GrabBlur merges and aggregates all sample-specific data. If a certain SNV is rare, the sample-specific information is replaced with the submitter identity. Generally, all data in GrabBlur are highly aggregated so that they can be shared with others while ensuring maximum privacy. Thus, it is impossible to reconstruct complete exomes or genomes from the database or to re-identify single individuals. After the individual information has been sufficiently "blurred", the data can be uploaded into a publicly accessible domain where aggregated genotypes are provided alongside phenotypic information. A web interface allows querying the database and the extraction of gene-wise SNV information. If an interesting SNV is found, the interrogator can get in contact with the submitter to exchange further information on the carrier and clarify, for example, whether the latter's phenotype matches with phenotype of their own patient.

## Background

Since the introduction in 2005, Next Generation DNA Sequencing (NGS) has been used successfully in numerous research projects [1]. Meanwhile, further technological advances have reduced the per base pair sequencing costs dramatically, thereby allowing more and more molecular diagnostics laboratories to screen the complete exome of individual patients with an apparently inherited disease for causative mutations [2]. Indeed, exome sequencing has already started to revolutionize diagnostic genetic testing [3][4]. However, pertinent data privacy law, the type of informed consent declarations used and limited genetic counseling resources bar sharing of high-resolution genetic data with third parties in most countries. From both a medical and a scientific point of view, this "locking" of data is hardly compatible with good professional practice. For instance, for a physician or geneticist it may be essential to know whether a particular mutation found in the genome of their patient has been found in another patient with a similar phenotype before. Related questions are also likely to arise in basic research projects on both monogenic and complex (i.e. oliogenetic) diseases.

## Tool description

We developed GrabBlur, a tool to collect and aggregate (i.e. "grab" and "blur") 'single nucleotide variants' (SNVs) linked to a specific trait or phenotype, and to share them with others by way of a public database while keeping individual samples unidentifiable. The database will not only help human geneticists to distinguish between benign variant findings and truly disease-causing mutations, but will also benefit genetic epidemiological research (i.e. case-control association studies) based upon large-scale SNV data.

In contrast to databases like ClinVar (http://www.ncbi.nlm.nih.gov/clinvar/) or the Human Gene Mutation Database (HGMD) [5], which only contain out-of-context information on genotype-phenotype associations, GrabBlur provides access to all SNVs detected in a given patient alongside the description of their specific phenotype. The Exome Variant Server (EVS) [6] provides about 2 million annotated SNVs of 6,500 individuals with heart-, lung- and blood-related diseases; more details are not specified. Through the straightforward aggregation of SNVs, it is not possible to find out, which SNV originated from which individual and phenotype. The EVS helps researchers excluding SNV candidates found in patients with monogenetic diseases, but it is not a resource to exchange genotypic and phenotypic data from other data sets, especially for Mendelian diseases where often the exact phenotypes are needed. Owing to this level of comprehensiveness, GrabBlur helps users not only to reckon known mutations, but also to validate newly found ones.

The most important feature of GrabBlur is the high level of anonymity ensured by its process of data aggregation. No conclusions as to the identity of a patient can be drawn even if the entire data stored for that individual are downloaded or the whole database is mirrored. It is possible neither to reconstruct a single patient genome nor to re-identify a patient from knowing their SNVs. Data is aggregated at the site of the submitter, i.e. behind their own firewall and under their responsibility for data protection. Hence, no identifying data leaves the submitter institution, and even if the data is "tapped" by an unauthorized person during upload to the database, a high level of privacy protection is maintained.

DNA sequence data are accepted by GrabBlur in standardized VCF format [7]. Additional information such as the phenotype or gender of a patient is stored in a separate "initialization file" (INI file format). Most of this information is optional and provision is at the discretion of the submitter. The following information may be recorded:

• **Trait**. A description of the disease of all patients in a GrabBlur set of samples (see below). Samples must be marked at least as 'patient' or 'healthy control'. (mandatory)

• **Phenotype**: GrabBlur uses Human Phenotype Ontology (HPO) terms [8] to classify phenotypes. Every phenotype can be ascribed an unlimited number of HPO terms. (optional)

• **Gender**: Gender of a single patient. (optional)

• **Platform**: DNA sequencing technology used. (optional)

• **Enrichment**: DNA enrichment kit used for sequencing. (optional)

• **PI**: Identity of principal investigator. (optional)

• **Contact details**: Identity, affiliation and e-mail address of the submitter (mandatory for upload, but optional release to public database)

**Figure 1:**
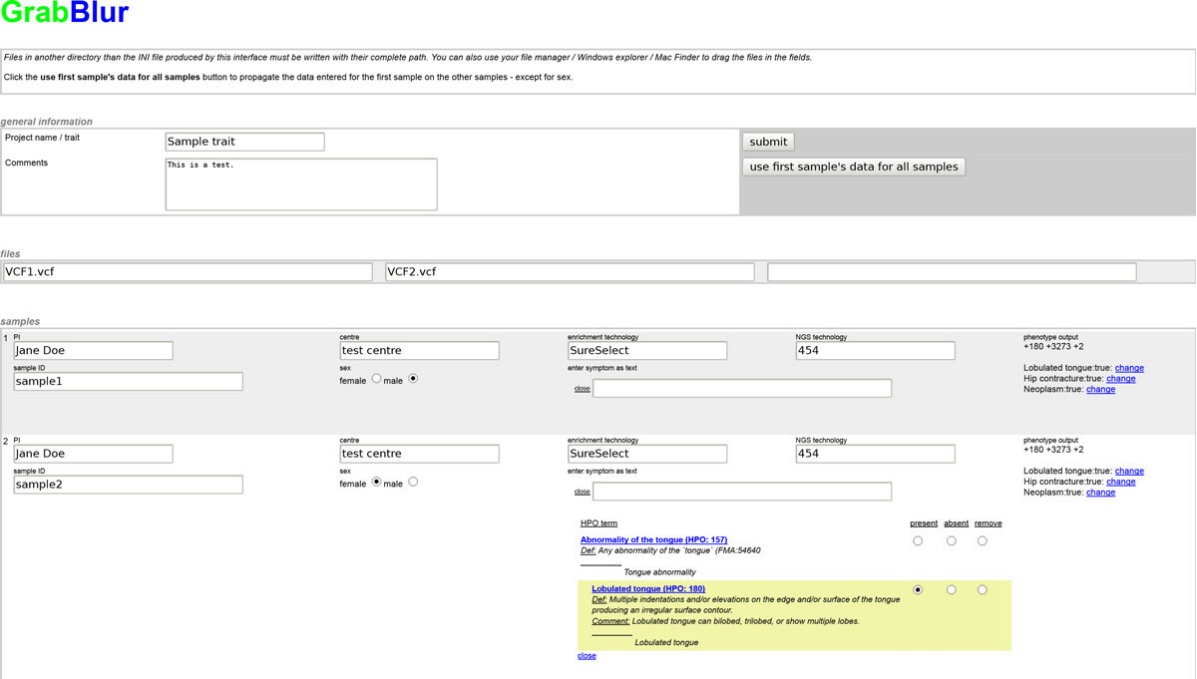
**Creation of the initialization file**. Screenshot of the interface to create the initialization files. The auto-completion procedure finds all HPO terms matching the user input. The chosen terms are then presented in a tree structure, with their definitions accompanied by parent and children terms.

To help users with the creation of the initialization file, we developed a web interface (Figure 1) to comfortably enter the required information, including sample ID and phenotype description. Although GrabBlur encodes phenotypes by a combination of HPO terms, users do not have to translate symptoms into numeric IDs. Instead, we employ an auto-completion procedure that finds all HPO terms matching the user input. The chosen terms are then presented in a tree structure, with their definitions accompanied by parent and children terms. This allows users to easily refine their description by choosing a more eligible term. In addition to marking symptoms as present, users can also identify particular symptoms as being absent to accentuate interesting characteristics of their patient.

On the project homepage, we also provide Perl scripts to read either a single VCF file or all VCF files contained in one directory and to directly submit filenames and sample IDs to this interface.

GrabBlur aggregates data in the following three steps:

1. Inspection of the additional information available for every patient

To prevent identification of a patient via the combination of different individual-specific informational items, these items must not be unique in the set of sample data provided to a third party. Every variant of a patient is associated with his meta-data. In case of uniqueness, the reconstruction of a patient's genome would be possible. At least two samples must have exactly the same phenotypes, same gender information etc.

In order to generate a sufficient level of ambiguity, samples with an identical set of HPO terms are combined in classes. If any other additional information is not sufficiently ambiguous, GrabBlur blurs it by deleting e.g. the gender or the platform-name.

2. Fragmentation of the SNV-data

In a second step, the SNVs of a sample are divided into sub-samples of different size. A list linking sample IDs and sub-sample IDs is stored in a encrypted and password-protected file at the submitter site. Encryption is accomplished by means of the Blowfish algorithm of OpenSSL [9]. Only the submitter themselves can open this file. This is needed, for example, to delete a sample from the database in case the patient withdraws the consent.

Each SNV of a sample is randomly assigned to a sub-sample. This assignment is not uniformly distributed because otherwise any group of linked sub-samples would contain an approximately equal number of SNVs, thereby allowing reconstruction of the complete sample. Therefore SNVs are assigned to a sub-sample with a differently weighted likelihood.

3. Blurring the genotype information for rare variants

In a third step, all rare variants of a sample are aggregated by replacing the sub-sample ID of a rare SNV by the contact information of the submitting institution. Since a patient can easily be identified by singletons (i.e. SNVs that have been detected only once), these and other rare SNVs in their exome are blurred. In the aggregation step the association between an SNV and all belonging sub-samples has been deleted. Only the trait and (if known) the submitting institution remain linked to the SNV. Hence, only common SNVs carry a sub-sample ID and, therefore, are associated with specific phenotype information.

The threshold for a variant to be considered rare is variable and depends upon the submitted data. It is calculated from the median of all SNV frequencies as

freq(SNV)=<1.5*med(frq)

Here, *freq(SNV) *denotes the frequency of the SNV irrespective of its genotype, and *med(frq) *is the median over all SNV frequencies in the sample set. We choose the median because it is robust against outliers, like in this case above-average number of singletons.

The default factor of 1.5 can be modified by the submitter to get a lower or higher aggregation level. Usually, with a default factor of 1.5, the threshold equals between 8 and 12 so that a data set must comprise at least 8 to 12 samples in order to provide additional information other than the contact address.

**Figure 2:**
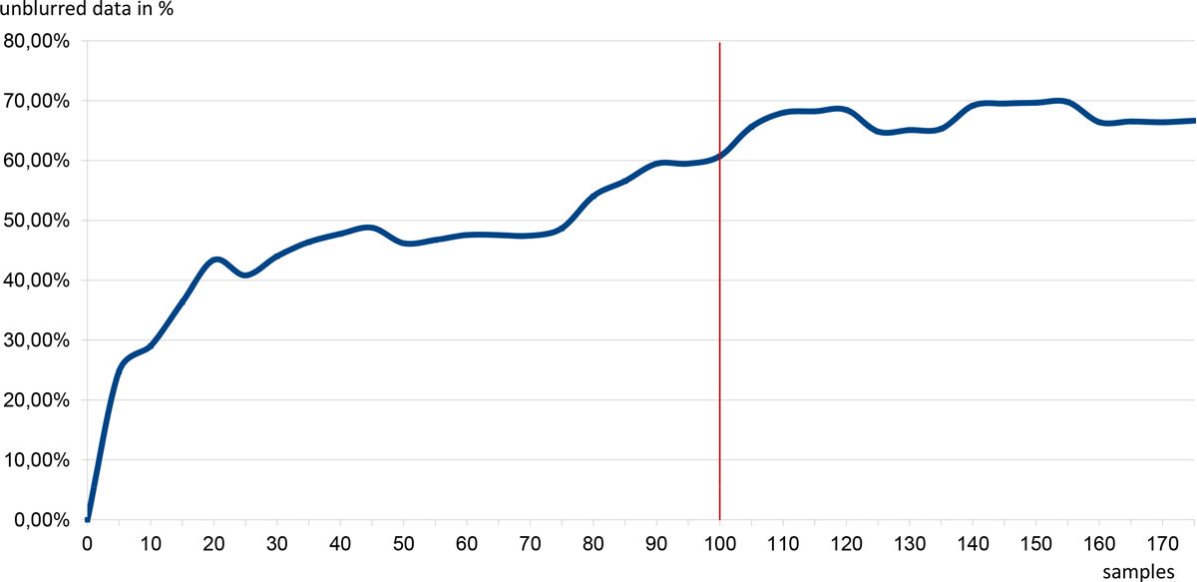
**Ratio of the unblurred data with various sample set sizes**. The ratio of unblurred data (SNVs where the genotype is not aggregated) in relationship to the amount of aggregated samples using the default median factor of 1.5. The part of unblurred data increases logarithmically to the number of aggregated samples. Through the blurring of the rare variants the portion of unblurred data is reaching a plateau of about 70%. Hence, a good minimum sample size is n = 100 so that as less genotype information as possible gets blurred. For blurred SNVs no genotype will be notified, only the contact data of the submitter will be named.

Figure 2 shows the ratio of unblurred data (SNVs where the genotype is not aggregated) in relationship to the amount of aggregated samples using the default median factor of 1.5. The part of unblurred data increases logarithmically to the number of aggregated samples. Through the blurring of the rare variants the portion of unblurred data is reaching a plateau of about 70%. Hence, a good minimum sample size is n = 100 so that as less genotype information as possible gets blurred.

**Figure 3:**
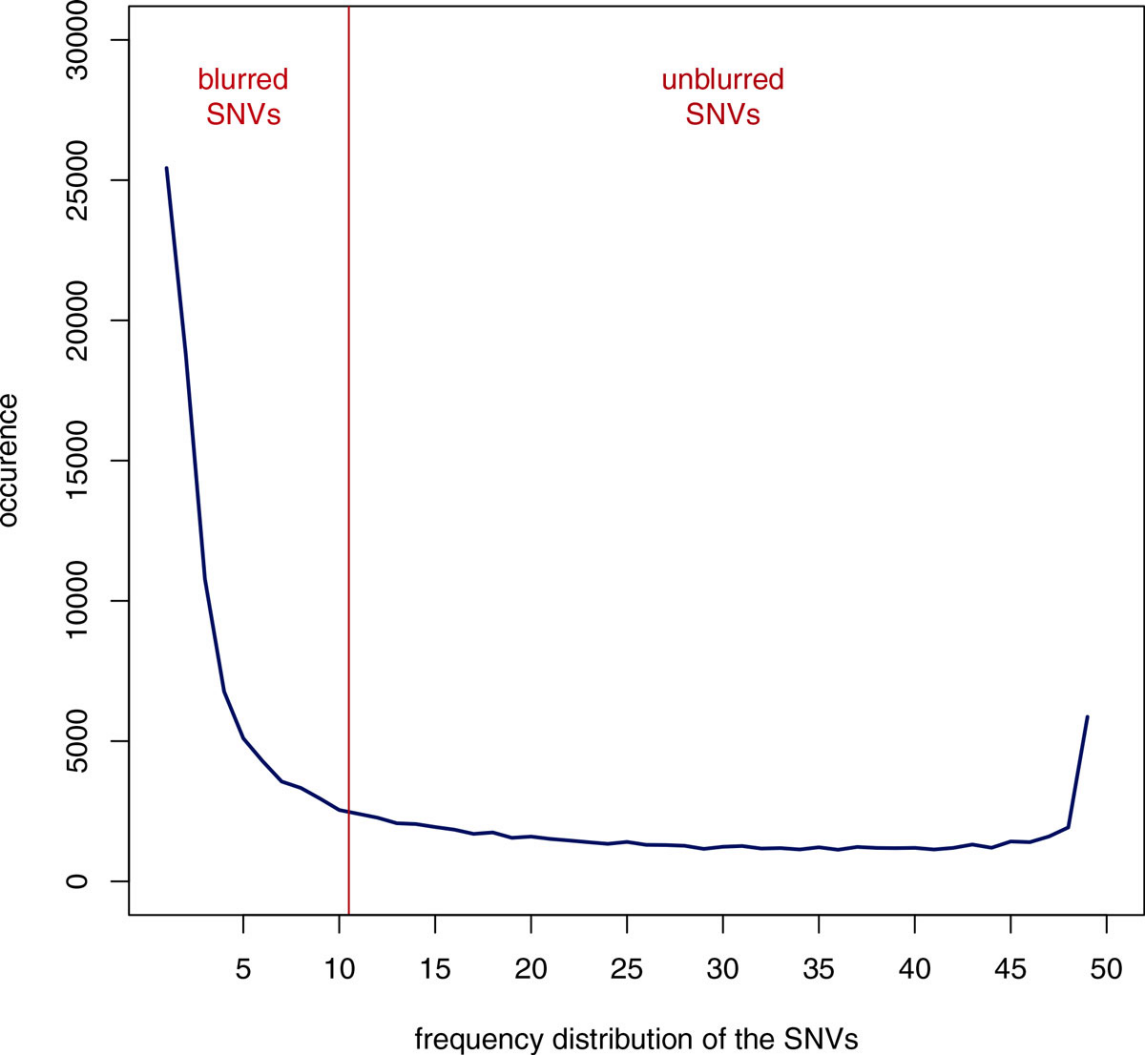
**SNV frequency distribution in a sample of 50 exomes**. Evidently, the proportion of singletons is 10 times higher than that of common SNVs. In the depicted example, the threshold frequency for rare variants was approximately 20% resp. 10 SNVs.

Figure 3 shows the frequency distribution of a sample set containing 50 randomly chosen exomes. As expected, the proportion of singletons is 10 times higher than that of common SNVs. In the given example, the threshold frequency for rare variants would be approximately 20% (or 10 occurrences).

## Aggregation quality

**Figure 4:**
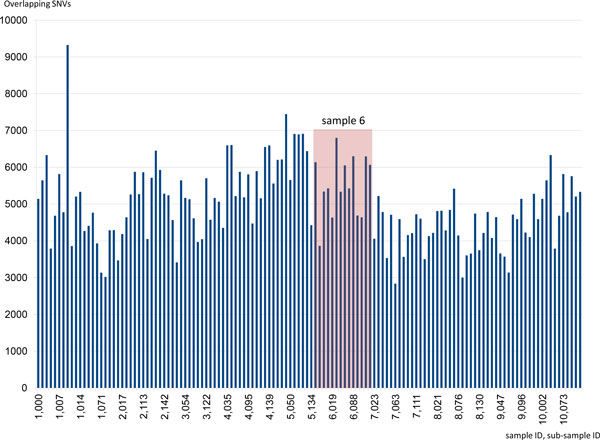
**Assessment of the aggregation quality**. To assess the aggregation quality of GrabBlur, a sample set of 10 individuals was "blurred" and compared to a non-aggregated sample of that set. For illustration purposes, the sub-samples were sorted according to the original sample IDs (sample ID, sub-sample ID on the X-axis). The corresponding individual is sample no. 6. The overlap between the sub-samples originating from this sample is not significantly higher than with the other samples.

To assess the aggregation quality and hence the level of ensured anonymity, a sample set of 10 individuals was blurred and compared to one randomly selected and non-aggregated sample of that set. For illustration, the sub-samples in Figure 4 were sorted according to the original sample IDs (sample ID, sub-sample ID on the X-axis). The selected individual is sample no. 6. It turns out that the overlap between a sample and its matching sub-samples is not notably larger than with other sub-samples. This is important if the data upload is intercepted or if the database itself gets compromised. Moreover, the aggregation also makes it impossible for an interested authority to identify an individual by comparing their own genetic data to the GrabBlur database (e.g. a law enforcement authority searching for a suspect).

## Data access

**Figure 5:**
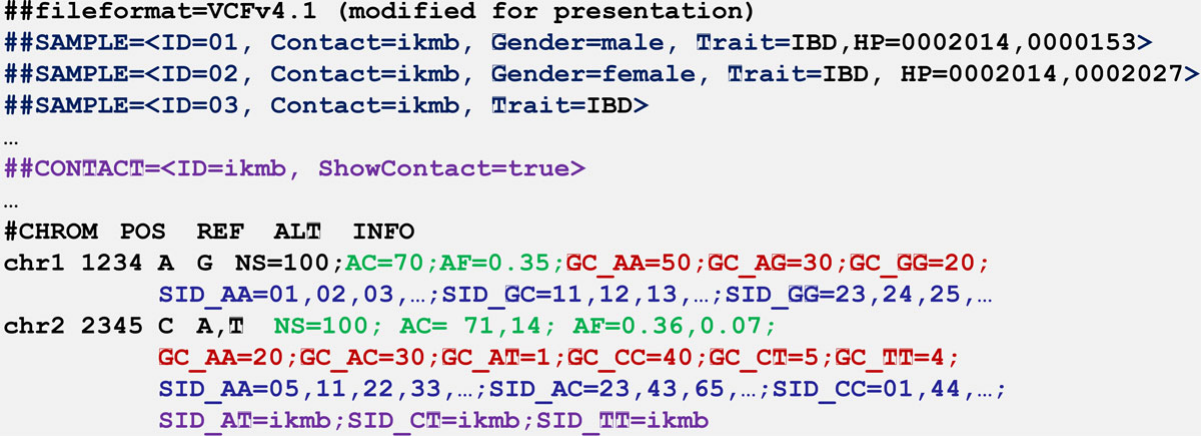
**Output file "aggregated VCF"**. GrabBlur writes the aggregated SNV data of all samples into a new VCF file. This figure shows a typical GrabBlur output of blurred SNVs. Some VCF information has been excluded, such as the dbSNP-ID and quality information, for explanatory purposes.

After the aggregation steps, the blurred data is written into a new VCF file at the submitter site (Figure 5) from where they are being uploaded to the public database. This process does not start automatically so that the submitter keeps control of the data they provide. After uploading, other registered users are able to retrieve information on the submitted SNVs and their associated phenotypes. To access the GrabBlur database, we developed a web front end (accessible at http://grabblur.ikmb.uni-kiel.de) that offers two main features:

(1) After registration, users can upload their data. The web front end allows the user to choose an aggregated VCF-file, which must have been created before using the blurring software described above. The front end sends the file to a client software running on the same server, which checks the file for consistency and potential corruptions and then transfers it to the database.

During the upload-process, every SNV is automatically functionally annotated using our in-house software tool *snp*Acts (http://snpacts.ikmb.uni-kiel.de). *snp*Acts identifies whether an SNV causes a protein coding substitution and which amino acid is affected using the gene annotations from CCDS [10] and RefSeq [11]. The amino acid changes in all iso-forms of the affected gene are classified and ranked in the following order: "nonsense" (most likely to be damaging), "readthrough", "start-lost", "splice site", "missense", "synonymous" (least likely to be damaging). To obtain more information for estimating whether an SNV is likely to be damaging, *snp*Acts also queries the Human Gene Mutation Database "HGMD" [5]. HGMD provides a database of comprehensive, in part manually curated data on human inherited disease mutations. Since this is a commercially available database, only an identifier from the HGMD database is named in *snp*Acts. All results of these annotations, including the highest ranked classification of the SNV, are stored in the database upon upload of the data.

**Figure 6:**
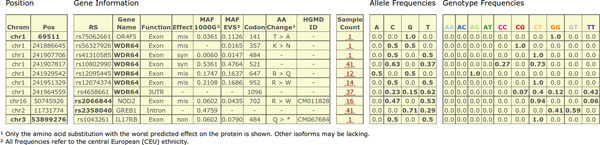
**Web front end - search results**. The figure shows an example search result for selected loci. The latter does not lie within a gene, so no additional gene information is provided. For the other two search terms the in-silico predicted gene function and effect of the SNV on the gene are provided. For all loci the number of samples in database with known genotype information is given - along with the frequencies for the genotypes and just the alleles.

**Figure 7:**
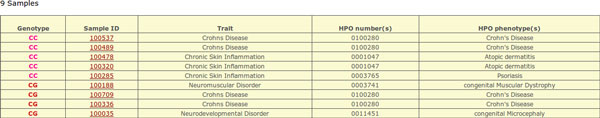
**Web front end - samples associated with one locus**. This view of the front end gives detailed information about the samples associated with one particular locus including the actual genotype, the trait (as an user definable term) and the HPO ID and terms as they were determined during the creation of the upload file.

**Figure 8:**
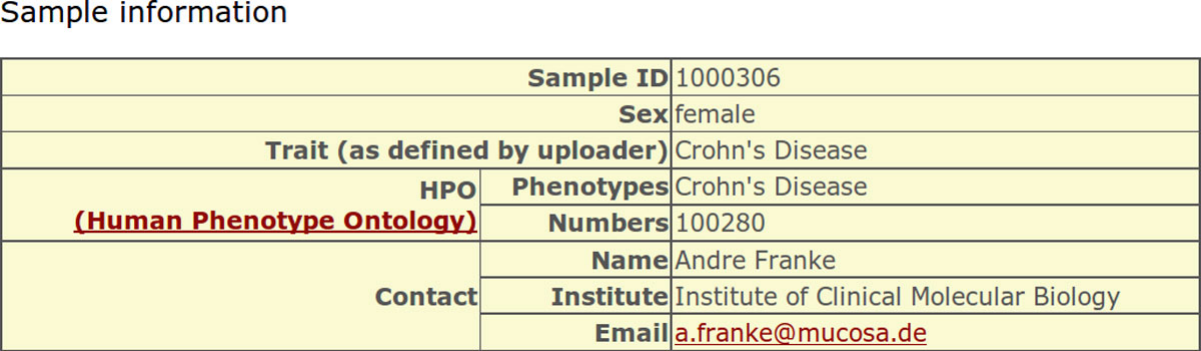
**Web front end - sample details**. In addition to the sample list (Figure 7) this detailed view provides the sex of the sample and the contact information provided by the uploader to get more information on the sample.

(2) All registered users are able to search the database for loci of interest using either the chromosomal position, the dbSNP IDs of known SNVs, gene symbols, or a protein position in combination with a gene symbol. The latter is particularly useful to identify potentially compound heterozygote samples. However, phase information needs to be retrieved from the submitter via re-contact. Registered users can also perform combined searches simultaneously looking for terms of different type (e.g. "chr1:13272" and "rs6605067" and "NOD2"), so the search result contains information about the locus, i.e. weather it is situated within a gene, the gene identifier and gene function, and how many samples in the database carry an SNV at this locus (Figure 6). The allele and genotype frequencies of every SNV over all samples in the database are displayed as well as publicly available allele frequencies from the 1000 Genomes Project [12] (phase1) and from the Exome Variant Project [6] (ESP6500SI-V2). The user can access further information for each of these sets of samples, if provided by the submitter, including the associated trait in the form of HPO terms (Figure 7). Additional information, like the submitter contact information or the sample gender (Figure 8), can be obtained also if provided.

## Implementation

The aggregation software was written in C++ on an Ubuntu Linux system. The runtime of the aggregation increases linearly with the amount of samples. The consumption of memory (RAM) increases logarithmically. On a desktop PC, a VCF file with 43,000 SNV was aggregated in less than 3 seconds using one core (Intel Xeon 4C, 2.0 GHz). The aggregation of 50 exomes with about 40,000 - 45,000 SNVs needs approximately 128 MB RAM and 130 sec. The aggregation of 150 exomes needs about 7 minutes with approximately 350 MB RAM.

The interface for the creation of the initialization files is programmed in Perl and uses JavaScript and AJAX to display the HPO terms retrieved from a PostgreSQL database.

The web interface for data access has been implemented using the Django web application framework [13] (v1.5.4) and the Python programming language [14] (v3.2.2). It is currently running on an Ubuntu Linux Server (12.04.3 LTS). The MySQL database containing the actual GrabBlur data is located on another server (with the same configuration) and is accessed using the respective built-in modules of Django and Python.

## Discussion

GrabBlur is a "light weight" tool to aggregate SNV data of thousands of samples with a specific trait or phenotype and to share the data with other via a public database. The main goal of GrabBlur, namely to keep each individual sample unidentifiable, was achieved by deleting other important information from individual exomes or genomes. For instance, all information of linked SNVs must be dropped to avoid the reconstruction of a given data set. But exactly this information is very valuable for scientific studies. For example, rare variant association analysis methods collapse rare variants into groups based upon, for example, the functional annotation of genomic regions. Whether GrabBlur can be used in such studies needs to be verified individually for each analysis method (for a review of methods, see [15]). However, GrabBlur is intended mainly to serve human geneticists who try to find more data on a variant and the phenotype of interest. The user-friendly GrabBlur web interface should inspire users to share their data and to use the tool for their own purposes. Although GrabBlur anonymizes the genetic data to a sufficient degree, a cautious user may want to use GrabBlur only behind their own firewall to handle aggregated information. While we encourage users to share their data, we also support such "internal" mirrors and provide instructions to set them up.

GrabBlur also has limitations that should not go unmentioned. For example, the system is not yet checking for duplicate uploads. It is thus possible that redundant data end up in the GrabBlur database. Moreover, the quality of an uploaded SNV may not have been adequately checked. Detailed quality data, as it can be generated using our previously reported tool pibase [16], would require that users also retrieve BAM files for their sequence data, run additional and standardized analyses. Moreover, the addition of the quality scores would significantly inflate the GrabBlur database. We rather prefer that submitter provide their contact details so that data users can enquire the quality of particular SNVs directly. The submitter may then go back to the raw data and use pibase, the Integrated Genomics Viewer [17] or other tools to assess the quality of the SNV in more detail. It is also possible with GrabBlur to ask submitters for additional details on the phenotype of a patient or for a detailed re-phenotyping based on new scientific findings.

## Competing interests

The authors declare they have no conflict of interests in relation to this SNP-SIG issue article.

## Declarations

The publication costs for this article were funded by the DFG Cluster of Excellence No. 306 „Inflammation at Interfaces".

This article has been published as part of *BMC Genomics *Volume 15 Supplement 4, 2014: SNP-SIG 2013: Identification and annotation of genetic variants in the context of structure, function, and disease. The full contents of the supplement are available online at http://www.biomedcentral.com/bmcgenomics/supplements/15/S4
